# Pain during walking and ascending stairs before hyaluronic acid injection was common in patients with knee osteoarthritis: a qualitative study

**DOI:** 10.3906/sag-2007-248

**Published:** 2021-04-30

**Authors:** Ömer ÖZKAN, Naila BABAYEVA, Şerife Şeyma TORGUTALP, Ömer Serkan KARA, Gürhan DÖNMEZ, Feza KORKUSUZ

**Affiliations:** 1 Department of Sports Medicine, Faculty of Medicine, Hacettepe University, Ankara Turkey

**Keywords:** Knee, osteoarthritis, qualitative study, injection, hyaluronan

## Abstract

**Background/aim:**

Knee osteoarthritis (OA) is a common pathology characterized by degeneration of the articular cartilage. The aim of the research was to ask patients how they decided to make the injection, what treatments they received, their complaints prior to and after the injection and how they feel at the moment, and whether they are currently exercising or not. Thus, to demonstrate the patients’ outcomes with their own expression.

**Materials and methods:**

A total of 92 knee OA patients completed semistructured interviews, which included six open-ended questions.

**Results:**

A total of 92 patients (66 female, 26 male) aged between 36 and 95 years (mean 65.511.14) were included. Before the injection, the majority of the OA patients had pain complaints when walking (72.8%) and stair climbing (70.7%). One to four years after intraarticular injection, 45.2% of patients felt a decrease in their complaints. The majority of patients did not consider diet and exercise as a treatment option. In addition, almost all patients declared that they decided on hyaluronic acid injection treatment with the physician’s recommendation.

**Conclusion:**

Pain during walking and stair climbing before hyaluronic acid injection was common in knee OA patients. Overall the patients felt a decrease in the symptoms after injection. Patients did not consider diet and exercise as a treatment option despite the recommendation by a physician.

## 1. Introduction

Progressive joint pain, deformity and limitation of movement are common clinical findings of osteoarthritis (OA) that may decrease the quality of life and may lead to morbidity and mortality [1, 2]. The knee joint is the most common site of OA among the peripheral joints and is retained the second after the spine in the entire body [3, 4]. Progression of OA can lead to total arthroplasty of the knee joint [5]. The intraarticular injection of hyaluronic acid (HA), also called viscosupplementation, is a nonsurgical approach to the treatment of OA and has become an increasingly popular treatment method in recent years [6–8]. 

Several studies related to the knee OA have found that HA injection is an effective and safe treatment for improving the functional status and decreasing pain, along with causing less significant side effects [7–10]. According to a Cochrane review, HA injection has a therapeutic benefit for pain during weight-bearing over placebo at 5 to 13 weeks postinjection [11]. On the other hand, other metaanalysis showed no significant results involving pain relief and functional improvement after the use of HA [12]. To better understand patients’ perception and outcome of the knee function after the treatment, patient-reported measures such as questionnaires are used widely in clinical and research practice [13]. More recent metaanalysis on this matter by Zhang et al. [14] found that platelet-rich plasma (PRP) injections were more effective in reducing pain than HA injections in knee OA patients at 6 and 12 months of follow-up assessed by the Western Ontario and McMaster Universities arthritis index (WOMAC) pain score questionnaire, whereas the visual analogue scales (VAS) questionnaire showed no significant difference at 3 and 6 months. On the other hand, in a double-blind randomized controlled trial of Cole et al., WOMAC measures did not show a significant difference between PRP and HA groups [15]. 

Different from this quantitative studies, the qualitative study attempts to find the meaning of the case through descriptions, experiences, and views of the participants [16]. Based on this, the researcher seeks to analyze the subjects’ history regarding topic of interest in and deduce in terms of meaningfulness and importance [17]. This form of research allows participants to express their views on their own terms. Mays and Pope [18] pointed out that this type of interview is a flexible and powerful tool and can open up new areas for research. Thus, the qualitative method was deemed as a useful tool in the measurements of the outcomes after HA injection, while it has a potential to develop the uncovered field, due to the lack of qualitative research in this area.

To our knowledge, patient-centered qualitative evaluation of the success of HA knee injection is based on very limited data. The aim of the research was, therefore, to evaluate patients’ perceptions, outcomes, and sports activity participation within 1–4 years after intraarticular HA injection, as a treatment option for knee OA. 

## 2. Patients and method

### 2.1. Design 

This study was designed based on the principles and methods of constructivist grounded theory [19]. Researchers’ goal was to obtain as much information as possible from the patients’ own words, to understand beliefs and actions from their perspectives and locate patients’ meanings within larger social structures and discussions in explorative qualitative research designs [20]. Data were obtained anonymously to ensure confidentiality. 

### 2.2. Sampling and data collection 

In this Turkish population-based qualitative designed study, 92 patients aged 36–95 years (mean 65.511.14), completed semistructured interviews. Included patients were individuals ≥35 years presenting with knee pain and Kellgren–Lawrence (KL) grade 1–3 primary knee OA diagnosed according to American College of Rheumatology (ACR) classification Criteria. Thirty-one patients (34%) had KL grade 1 OA, 32 (35%) grade 2, and 29 (31%) grade 3. KL grade was determined by a physician experienced in Kellgren–Lawrence (KL) radiographic classification of knee osteoarthritis. Patients were free of any hearing or speech impairment. Other inclusion criteria were: pain > 3 months, mean pain severity ≥2 on the numeric rating scale (NRS), Kellgren and Lawrence (K&L) grade I to III in medial and/or lateral compartment as well. Patients with injuries of the knee, also who had knee pain referred from the low back, KL grade 4 OA, case with history of previous intraarticular injection, severe hip OA, dermatologic knee disorders, chondrocalcinosis, nonknee-related regular analgesic use, inflammatory arthritis, allergy to HA components, planned pregnancy or lactation, daily oral steroid therapy, poor general health, conditions interfering with functional assessments, alcoholism, malignancy, age > 80 years, history of the knee surgery, along with psychiatric disorders and dementia were excluded. 

HA injections to participants were carried out every week for 3 weeks. A semistructured interview with six open-ended questions (Table 1) was developed by the research team, which consisted of experienced clinicians, to explore personal experiences and perspectives of patients after HA injections. They were conducted with patients within 1–4 years, 1st year as the earliest and 4th year as the latest period, after intraarticular HA injections. Interviews were conducted during routine outpatient clinic visits of patients, and then written down by two interviewers. Each interview with patients was audiotaped and transcribed verbatim with the average length of the interviews about 50 min. In the literature, semistructured interview refers to collect data by analyzing patient’s speech and its content [18]. 

**Table 1:** Six open-ended questions.

1. What was your complaint prior to injection?
2. Which treatments were applied prior to the injection?
3. How did you decide to make an injection?
4. What did you feel during first 3 days after injection?
5. How do you feel yourself right now?
6. Did you return to exercise?

### 2.3. Analysis 

Open coding, axial coding and developing a core category that clarified the central matter of the data was used for data analysis [19–21]. All written answers were scanned and qualitative data analysis was conducted with each transcript individually read line by line by two researchers. Subsequent steps of the analysis consisted of identification of the relevant texts, fragmentation of the text in parts of meaning and developing code from the obtained data. Qualitative responses were coded and converted into quantified data by this method. These results were afterwards discussed with the whole research team regarding definitions and application of the codes to ensure validity. To enhance the trustworthiness of the data, an audit trail documenting the processes of data reduction and analysis was maintained. 

## 3. Results

A total of 92 patients (66 female, 26 male) aged between 36 and 95 years (mean 65.5 11.14) were included. When patients were asked about their complaints prior to injection, the majority of them responded that they had complaints about the pain during walking (72.8%) and stair climbing (70.7%). Responses of other patients were functional limitations (41.3%), waking up with pain at night (18.5%), unable to sit on the knees (16.3%), crepitations (14.1%), knee locking (12%) and sensation of “giving-way” (3.3%). A small minority of patients gave as an answer pain in cold weather and while standing, burning sensation, feeling of knee deformation and feeling like a sharp piece of glass is cutting into the knee (Table 2).

**Table 2:** Answers of the participants to open-ended questions.

N = 92	N*	%
What was your complaint prior to injection?		
Pain during walking	67	72.8
Pain during ascending the stairs/hill	65	70.7
Functional limitations	38	41.3
Waking up with pain at the night	17	18.5
Unable to sit on the knees	15	16.3
Crepitations	13	14.1
Knee locking	11	12
Sensation of “giving away”	3	3.3
Other	16	17.4
Which treatments were applied prior to injection?		
Oral/topical NSAIDs	38	48.1
Physical therapy	32	40.5
Injection	29	36.7
Exercise	12	15.2
Oral chondroprotective agents	9	11.4
Other	7	8.9
How did you decide to make an injection?		
Physician’s recommendation	83	90.2
Friend/community	14	15.2
Complains	3	3.3
Other	6	6.5
What did you feel during first 3 days after injection?		
Did not feel any difference	52	56.5
Felt more comfortable	23	25
Pain increased	10	10.9
Do not remember	8	8.6
How do you feel yourself right now?		
Complaints decreased	42	45.6
Do not have any complaints	18	19.5
Complaints remained unchanged	16	17.4
At the beginning complains decreased/gone, but now recrudescence in complaints	16	17.4
Complaints increased	1	1.1
Did you return to the exercise?		
None	42	47.7
Walking	36	40.9
Home exercises	9	10.2
Swimming	8	9.1
Running	3	3.4

In response to the second question, patients reported that they had oral and/or topical NSAIDs (48.1%), physical therapy modalities (40.5%), injection (36.7%), exercises (15.2%), oral chondroprotective agents (11.4%) and other treatments like cold bandage, cold pack and alternative medicine (8.9%) (Table 2).

When the subjects were asked how they decided to receive an intraarticular injection, it was shown that 90.2% of patients decided to have an injection with the physician’s recommendation, 15.2% of patients decided under the influence of their friends and others and 3.3% of patients decided because of their constant pain. According to the patients, other reasons for intraarticular injection were individual explorations such as a search on the Internet and considering the injection as the last option before the operation (6.5%) (Table 2). 

56% of those surveyed reported that after intraarticular injection, they felt no difference, where 25% of them felt themselves more comfortable. Only 10.9% of the respondents declared that their pain increased after the HA injection, and 8.6% do not remember how they felt during first 3 days after intraarticular injection (Table 2).

In order to obtain more information regarding side effects after HA injections, the fourth question was therefore extended. The majority of respondents (72%) felt none of the following symptoms: joint warmth, pain, fever or local erythema (Table 2).

One to four years after the intraarticular injection, 45.2% of patients felt a decrease in their complaints, 19.6% of patients had no complaints at all, 17.4% of patients did not feel any changes, whereas 17.4% of patients are currently observed relapse after initial symptoms’ reduction, and the remaining 1.1% of patients whose complaints have increased (Table 2).

After intraarticular injection, 40.9% of patients resumed walking, 10.2% of patients resumed to home-based exercises and 9.1% of patients resumed to swimming on a weekly basis, whereas 47.7% of patients did not return any type of exercise.

## 4. Discussion

The most common form of arthritis, along with the fastest growing reason of disability in the world, is OA [22]. About 18% of women and 9.6% of men over 60 years worldwide experience a symptomatic OA, and 25% of them do not perform everyday physical activities [23]. Intraarticular HA injections have become an increasingly popular treatment method in OA, in recent years due to their effectiveness and safety issues [6–8]. To the best of our knowledge, this is the first study with a qualitative evaluation of patients’ perception who previously underwent HA injection in the knee joint. 

The values of diseases are reflected in the personal experience of people describing their conditions [24]. According to The American College of Rheumatology, there are some key symptoms for osteoarthritis, including morning stiffness lasting 30 min, crepitus on motion, bony tenderness and bony enlargement [25]. Patients’ responses in this study are in line with these criteria. In our study, when the subjects were asked about their main complaints, 41.3% of them answered that they have functional limitations and 14.1% have crepitations, along with 17.4% of other complaints including bony enlargement. The majority of the respondents declared that they experience pain during walking (72.8%) and ascending the stairs (70.7%). 

Treatment options for OA are individualized for the patients’ needs and preferences in order to provide high-quality care for relieving these symptoms and improve quality of life [26]. According to the guideline of OA Research Society International (OARSI), that was published in 2014 [27], land- and water-based exercises, strength training, and weight management are the core treatments applicable to all individuals. Along with the aforementioned, additional pharmacological interventions can be advised, structured on the characteristics of the individual patient. When our patients were asked about treatments that were applied prior to injection, 48.1% took nonsteroidal antiinflammatory drugs (NSAIDs) and 40.5% proceeded to physical therapy. In a metaanalysis of Bannuru et al. [28], where the data regarding the efficacy of HA in comparison with NSAIDs for knee OA was obtained, declared that there was no significant difference between HA and NSAIDs in continuous follow-up at 4 and 12 weeks. However, given the more favorable safety in favor of HA over NSAIDs, the authors stated that HA might be an alternative to NSAIDs for knee OA, especially for elderly patients with a high risk of systemic side effects. More recent pieces of evidence suggest that various forms of exercise have positive effects on pain and joint function for OA patients [29,30]. However, only 9.4% of those surveyed reported that exercise program was recommended as a treatment in our study. These findings are consistent with previous reports on low-rate recommendation of exercise, noncompliance to exercise therapy and lifestyle counseling, and warrant further investigation to increase this low level of adherence [31,32]. The most appropriate knee OA management, including exercise therapy, should be designed according to a patient-centered approach.

When patients are facing serious illnesses, which can cause changes in body image and lifestyle activity, it is important to understand what influenced the patients regarding the decision-making process in treatment options. When the subjects were asked what influenced their decision to undergo HA injection of the knee joint, 90.2% said that the decision to choose a treatment option was proposed on the recommendation of the doctor. This result is in conformity with previous studies, showing the role of physician on decision making process [33]. It has been reported that HA is most frequently prescribed by physicians to patients with early-stage (82%) or mid-stage (82.8%) OA [34]. To our knowledge, this is the first qualitative designed paper addressing the role of clinician’s in choosing the use of HA for the treatment of knee OA. It is important to note that physicians have a potential influence regarding treatment recommendations that influence the choice of patients. 

The recommendations of the European League against Rheumatism (EULAR) show that there is evidence to support the effectiveness of HA in accordance with the level 1B indications for both pain reduction and joint functional improvement of the knee joint [35]. However, the expected effect can be obtained within a few months, rather than within a few weeks, as with the use of a steroid. Patients’ responses regarding their well-being during first 3 days after intra-articular injection, 56.5% of those surveyed reported no difference, where 25% of them felt themselves more comfortable. Only 10.9% of the respondents declared that their pain increased after the HA injection and 8.6% don’t remember how they felt during first 3 days after intraarticular injection. 

In order to get more information regarding side effects after HA injections, the aforementioned question was therefore extended and the 71% of respondents felt none of the following symptoms: joint warmth, pain, fever or local erythema. These results were similar to a systematic review and metaanalysis of Millers et al.[36], where the safety and efficacy of US-approved HA knee injections were randomized with saline controls, found no statistical difference regarding serious adverse effects between these two groups in patients with knee OA. Some adverse effects such as increased rate of flare [37], granulomatous inflammation [38] and a few local infections (like septic arthritis) have also been reported. A recent systematic review found no difference in the side effect rate between single injections of HA and placebo [39]. In our study, 21.7% of patients felt pain, fever or erythema, and none of our patients described any serious adverse effects like septic arthritis. The Cochrane review of 2014 showed that viscosupplementation for the knee OA provides pain relief and improved physical function with a low risk of harm [40].

Many researchers have studied the efficacy of HA injection in the treatment of the knee OA in long-term. According to the recent analysis of US-approved HA injections showed a better treatment effect compared to preinjection values from 4 weeks to 26 weeks for pain and knee function, in comparison with placebo [36]. In the study of Miltner et al.[41], where patients underwent HA injection, showed improvement at VAS and maximum peak-torque. In 2010, Chevalier et al.[42], stated that a single 6-mL intraarticular injection of Hylan G-F 20 was safe and effective in providing statistically significant and clinically relevant pain relief over 26 weeks, with a modest difference versus placebo. Our study is in line to these studies, where we found that 45.6% of patients felt the decrease in their complaints, 19.5% of patients currently do not have any complaints, and 17.4% of patients had at the beginning complaints decreased and even gone, but now recrudescence in complaints. DeCaria et al.[43] reported no difference in gait velocity compared with placebo, but they found that patients treated with HA had improvements in WOMAC scores for pain, stiffness, and physical function. 

Our study has several limitations including, prominently, the fact that the respondents were patients of the same hospital, and hence the results cannot be applied to the general population. Therefore, future studies with a larger sample with knee OA should be recruited. Secondly, there were difficulties in translating the patients’ interviews from Turkish to English in the concept of research results, even though the translation was performed by a professional translator who did not interview the subjects in this study. In consequence, the researchers repeatedly discussed the accuracy of the translation, so that the basic concepts were not lost in the translation. Therefore, future studies are needed to overcome these issues. 

In conclusion, we would like to mention that despite all the limitations, this research, to our best knowledge is the first qualitative study regarding outcomes before and after HA injection. Our study identified main complaints of the patients before injection, which were following, the pain when walking and stair climbing. Most of the patients underwent treatments prior to HA injections; however, many patients didn’t consider diet and exercises as a treatment option. Along with the aforementioned, our findings showed that choosing HA as a treatment option was proposed on the recommendation of the doctor. Therefore, physicians should improve their relationship with the patient by providing adapted and formalized information to patients regarding the efficacy of treatment strategies, adapting more approved guidelines and therapeutic approaches, which are the main factors of symptoms’ improvement in OA.

## Informed consent

All the participants gave written informed consent prior to the study and this study was conducted in accordance with the Declaration of Helsinki. The protocol was approved by the Ethics Committee of Hacettepe University (Decision number: KA-180014).
